# Synthesis and Characterization of P-PPD-Ph-Conjugated Flame Retardant

**DOI:** 10.3389/fchem.2022.956322

**Published:** 2022-07-18

**Authors:** Junzhuo Sun, Daohai Zhang, Ke Wei, Fang Tan, Min He, Dongmei Bao, Shuhao Qin

**Affiliations:** ^1^ School of Chemical Engineering of Guizhou Minzu University, Guiyang, China; ^2^ Polymer Composites Engineering Research Center of Guizhou Minzu University, Guiyang, China; ^3^ National Engineering Research Center for Compounding and Modification of Polymer Materials, Guiyang, China

**Keywords:** conjugated flame retardant, poly(lactic acid), structure, thermal performance, rheological properties

## Abstract

The conjugated flame retardants have rarely been studied. A conjugate flame-retardant 4, 4'-{1″, 4″-phenylene-bis [amino- (10‴-oxy10‴-hydro-9‴-hydrogen- 10‴λ^5^-phosphaphenanthrene-10″-yl)-methyl]}-diphenol (P-PPD-Ph) was synthesized and added into the polylactic acid (PLA) matrix. The P-PPD-Ph-conjugated flame-retardant structure was tested by FTIR, ^1^H, and ^31^P NMR analysis. The thermal and rheological properties of PLA/P-PPD-PH-conjugated flame-retardant composites were investigated. The results showed that P-PPD-Ph-conjugated flame retardant affects PLA/P-PPD-PH-conjugated flame-retardant composites for promoting the formation of a carbon layer when the P-PPD-Ph-conjugated flame-retardant content was 15% and the residual carbon ratio for PLA/P-PPD-PH-conjugated flame-retardant composites increased by 4.2%.

## Introduction

Polylactic acid (PLA) is an aliphatic polyester derived from renewable agricultural resources such as corn, rice, wheat, and sugarcane, and PLA is considered a promising material for reducing environmental pollution and solid waste disposal problems (Lin et al., 2018). PLA is widely used in medicine, construction, automotive interiors, electronic components, household goods, and transportation (Hou, et al., 2018; Jia, et al., 2018; Long, et al., 2019). As PLA is more flammable than polyethene and polypropylene, it requires flame-retardant treatment in textile, automotive, and electronic product applications (Zhang, et al., 2019; Mincheva, et al., 2020). Currently, 9,10-dihydro-9-oxa-10-phosphaphenanthrene-10-oxide (DOPO) is usually added to improve the flame-retardant performance of PLA composites. DOPO is a cyclophosphamide with a diphenyl structure, which has high thermal stability, good oxidation resistance, and water resistance (Yu, et al., 2014). DOPO shows excellent flame-retardant performance in materials (Li, et al., 2019). However, the flame-retardant effect of pure DOPO added to PLA cannot meet the production needs, so many DOPO derivatives are used to improve the flame-retardant performance of composites. Yu Tao group synthesized a diacid derivative (DOPO-MA) containing maleic acid (MA) and phosphate-based compound (DOPO-ICN) containing 1, 6-hexane diisocyanate (HDI). The results showed that DOPO-MA and DOPO-ICN improved the compatibility of PLA with jute, and DOPO-MA and DOPO-ICN gave jute/PLA composites better flame-retardant performance than DOPO(Yu, et al., 2017a; Yu, et al., 2017b). Niu et al. (2020) prepared a new flame-retardant hexa-(DOPO-hydroxymethylphenoxy-dihydroxy biphenyl)-cyclotriphosphazene (HABP-DOPO) by bonding DOPO with cyclotriphosphazene. The results showed that for the PLA mixture containing 25 wt% HABP-DOPO, the LOI value reached 28.5%, and UL-94 could pass v-0. The flame retardancy of the PLA/HABP-DOPO blend was significantly improved.

In this work, a conjugated flame-retardant 4, 4'-{1″, 4″-phenylene-bis [amino- (10‴-oxy10‴-hydro-9‴-hydrogen-10‴λ^5^-phosphaphenanthrene-10″-yl)-methyl]}-diphenol (P-PPD-Ph) was synthesized. The structure of properties of P-PPD-Ph-conjugated flame retardant was investigated. Also, the combustion behavior and rheological properties of PLA/P-PPD-PH-conjugated flame-retardant composites were investigated.

## Materials and Methods

### Materials

9,10-Dihydro-9-oxa-10-phosphaphenanthrene-10-oxide (DOPO) was purchased from Huawei Ruike Chemical Co. Ltd. (Beijing, China); PLA (DGEBA, commercial name: E-51) was purchased from Wuxi Diaisheng Epoxy Co. Ltd. (Wuxi, China); p-phenylenediamine was purchased from Kemiou Chemical Reagent Co. Ltd. (Tianjin, China); ethanol was purchased from Tianjin Chuandong Chemical Reagent Factory (Tianjin, China); and 4-hydroxybenzaldehyde was purchased from Kemiou Chemical Reagent Co. Ltd. (Tianjin, China).

### Synthesis of the Imine-Containing Compound

The imine-containing compound was synthesized according to the literature procedure (Sun and Yao, 2011). Briefly, p-phenylenediamine (0.10 mol, 10.814 g), 4-hydroxybenzalaldehyde (0.20 mol, 24.424 g), and 200 ml ethanol were added into a 500-ml round glass flask equipped with a condenser and a stirrer. The reaction mixture was stirred at 50°C under nitrogen conditions for 2 h; the reaction mixture then became thick because of the precipitation of the reaction product. Then, the mixture was cooled down to room temperature. The yellow precipitate was filtered and washed twice with ethanol and then dried at 80°C in a vacuum oven for 8 h. After drying, light yellow crystals of 31.01 g (88% yield) were obtained.

### Synthesis of the P-PPD-Ph-Conjugated Flame Retardant

**FIGURE 1:**
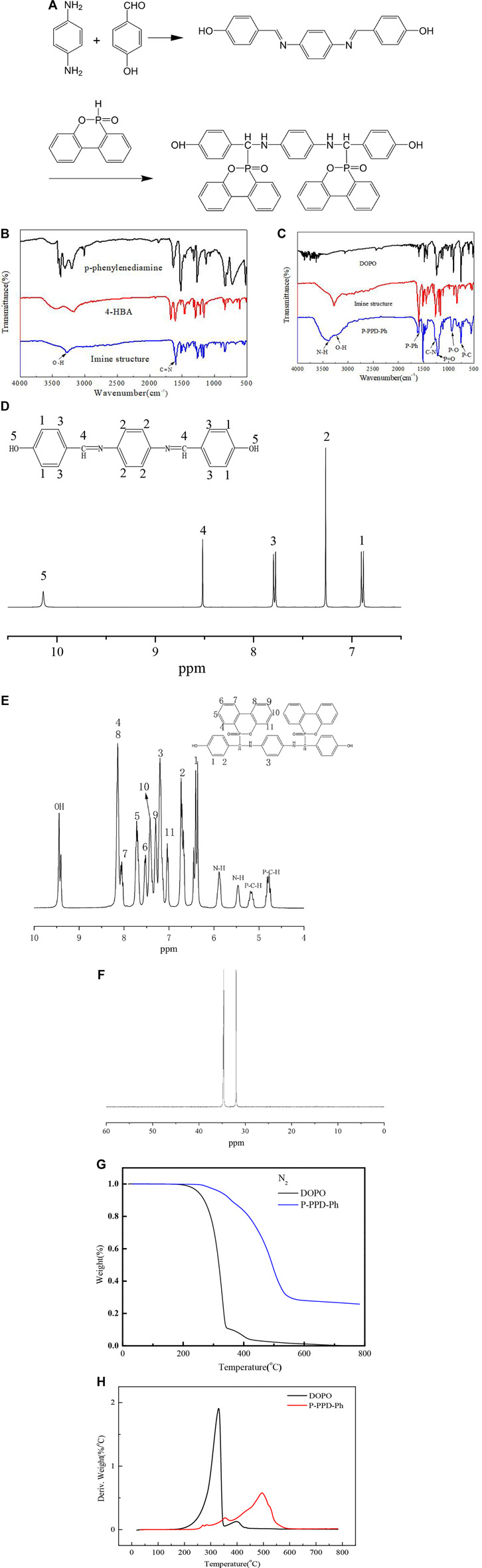
**(A)** Synthetic roadmap of P-PPD-Ph; **(B)** FTIR spectrum of the imine-containing compound and **(C)** P-PPD-Ph-conjugated flame retardant; **(D)** ^1^H NMR spectrum of the imine-containing compound and **(E)** P-PPD-Ph-conjugated flame retardant; **(F)** ^31^P NMR spectrum of P-PPD-Ph-conjugated flame retardant; **(G,H)** thermal analysis of the synthesized P-PPD-Ph-conjugated flame retardant under nitrogen (N_2_) atmosphere.

The P-PPD-Ph-conjugated flame retardant was synthesized according to the literature procedure (Sun and Yao, 2011). Briefly, the imine-containing compound (0.10 mol, 31.6 g) was synthesized according to 2.2, DOPO (0.20 mol, 43.237 g), and 300 ml of ethanol was added into a 500-ml round glass flask equipped with a condenser and a stirrer. The reaction mixture was stirred at 50°C for 10 h. Then, the mixture was cooled down to room temperature. The yellow precipitate was filtered and washed twice with ethanol and then dried at 80°C in a vacuum oven for 8 h. After drying, light yellow crystals of 31.61 g (88% yield) were obtained. The synthetic roadmap is shown in Figure 1A.

### Preparation of PLA/P-PPD-Ph-Conjugated Flame-Retardant Composites

PLA, chain extender, and P-PPD-Ph were dried for 4 h under vacuum at 80°C before use. PLA, epoxy chain extender (4 wt‰), and P-PPD-Ph (0, 5, 10, 15 wt%) were mixed uniformly. The model of the epoxy chain extender is ADR-5481, and its structural formula is provided by the manufacturer. Then, the mixture was extruded using a twin-screw extruder (CTE 35, Coperion Keya Machinery Manufacturing Co., Ltd., China) at the temperature of 180–200°C and screw speed of 300 rpm. The extruded pellets were then molded into samples for testing with an injection molding machine (CJ80MZ2NCII, Zhende Plastic Machinery Factory, China) at 180–200°C. During the reaction, the epoxy chain extender plays the role of the “bridge” to make P-PPD-Ph react with PLA.

### Characterization of the P-PPD-Ph-Conjugated Flame Retardant

The purity and structure of P-PPD-Ph-conjugated flame retardant were confirmed by FTIR, ^1^H, and ^31^P NMR analysis. FTIR spectroscopy was performed using a Nicolet 6700 spectrometer (Nicolet Instrument Company, United States of America) and ^1^H NMR spectrum was obtained with an Ascend 400 (Bruker BioSpin AG, Switzerland) using DMSO-d6 as a solvent with TMS as the internal standard. Also, the ^31^P NMR spectrum was obtained with an Ascend 400 (Bruker BioSpin AG, Switzerland) using DMSO-d6 as a solvent with H_3_PO_4_ as the internal standard.

Thermal analysis (TG) was conducted in a Q50 thermal gravimetric analyzer (TA, United States) at a heating rate of 10^°^C/min and under nitrogen conditions. Approximately, 5 mg of sample was weighed and placed in an aluminium ceramic with a gas flow of 60 ml/min.

Rheological measurements were performed on an advanced rheometric expansion system (ARES, TA Instrument, United States) using the parallel plate mode. The measurements were conducted at 200 °C.

The cone calorimeter test was conducted using an FTT cone calorimeter (UK) in accordance with ISO 5660-1 standard. The specimens were prepared with sizes of 100 * 100 * 6 mm^3^ and tested under a heat flux of 50 kW/m^2^. Each measurement was performed twice, and the results were averaged.

## Results and Discussion

### FTIR and NMR Analysis of the Imine-Containing Compound and P-PPD-Ph-Conjugated Flame Retardant and Thermal Analysis

An instrumental analysis of the powder was carried out by FTIR spectroscopy and ^1^H and ^31^P nuclear magnetic resonance. Figures 1B,C show the FTIR spectrum of the imine-containing compound (FTIR absorption: C=N 1605 cm^−1^, OH 3274 cm^−1^) and the FTIR spectrum of the P-PPD-Ph-conjugated flame retardant (FTIR absorption: N-H stretching 3,382 cm^−1^, O-H 3230 cm^−1^, P-Ph 1,599 cm^−1^, C-N 1277 cm^−1^, P=O 1213 cm ^−1^, and P-O 935 cm ^−1^).

The 1H HMR spectrum of the imine-containing compound is shown in Figure 1D, *δ* = 6.88(H1), 6.90(H1), 7.26(H2), 7.77(H3), 7.79(H3), 8.51(H4), and 10.13(H5). Also, the ^1^H HMR spectrum of P-PPD-Ph-conjugated flame retardant is shown in Figure 1E, ^1^H HMR, 4.74–4.84(P-C-H′), 5.12–5.22(P-C-H), 5.46(NH′), 5.88(NH), 6.26–6.45(1, 1′), 6.67–6.73(2,2′), 7.04(11, 11′), 7.20(3,3′), 7.29(9, 9′), 7.42(10, 10′), 7.53(6,6′), 7.68–7.72(5, 5′), 8.02–8.07(7, 7′), 8.10–8.14(4, 4′ 8, 8′), 9.40(OH), and 9.44(OH’). Furthermore, P-PPD-Ph exhibits two peaks at 31.64 and 34.66 ppm in the ^31^P NMR spectrum (as Figure 1F shows), and this is because there are isomers in the P-PPD-Ph-conjugated flame-retardant structure, and this is consistent with the literature reported by Sun and Yao, (2011). The ^1^H of P-PPD-Ph-conjugated flame retardant further demonstrates the presence of isomers in the P-PPD-Ph-conjugated flame-retardant structure.

The initial thermal degradation of the flame retardant occurs before the polymer decomposes, releasing the flame-retardant segment, thereby inhibiting the decomposition of the polymer matrix. The thermal degradation of P-PPD-Ph- conjugated flame retardant was investigated by TG under an N_2_ atmosphere, and the results are shown in Figures 1G,H, and the corresponding data are shown in Table 1. The initial decomposition temperature (temperature after 5% decomposed) (T_−5%_) of P-PPD-Ph-conjugated flame retardant was 74°C higher than the initial decomposition temperature (T_−5%_) of DOPO, and the carbon residue at 800°C increased by 25.8%, which may be due to the reason that P-PPD-Ph-conjugated flame retardant contains more aromatic groups and cage effect formed by flame-retardant particles during combustion (Fina, et al., 2014), and this finding indicates that aromatic groups promote the formation of more residues for DOPO derivatives (Long, et al., 2017). But the initial decomposition temperature of P-PPD-Ph-conjugated flame retardant was lower than that of some other DOPO derivatives; this is because the thermal stability of the DOPO derivative is related to the electron density of the C atom adjacent to the P atom. The lower the electron density was, the lower the thermal stability of the carbon was, and as a result, the thermal stability of DOPO derivatives is reduced (Lin, et al., 2010). The more electronegative N atom in P-PPD-Ph-conjugated flame retardant lowers the electron density of C, so the thermal stability of P-PPD-Ph-conjugated flame retardant is lower than that of some bridged DOPO derivatives containing only P-C bonds (Wang, et al., 2017).

**TABLE 1:** Data derived from TG analysis.

Sample	T_-5%_ (^°^C)	T_max_ (^°^C)	The value at T_max_ (_%/_^°^_C_)	Residue (%)
DOPO	250	330	1.9	0
P-PPD-Ph	324	494	0.6	25.8

### Linear Rheological Behavior of PLA/P-PPD-Ph-Conjugated Flame-Retardant Composites

Melt flow behavior for a material was usually studied by rheology, and the viscoelastic behaviors of the testing samples were investigated by rheology testing to illustrate the flame mechanism of P-PPD-Ph-conjugated flame retardant in PLA. The PLA/P-PPD-Ph-conjugated flame-retardant composites have lower storage modulus (G′), loss modulus (G″), and complex viscosity (η*) parameters than the PLA materials without flame retardants. Also, from the view of η*, the composites exhibit Newtonian-like fluid behavior, and this may be because the addition of P-PPD-Ph-conjugated flame retardant provides a larger free volume for PLA/P-PPD-Ph-conjugated flame-retardant composites, lowering the van der Waals force of the PLA, thereby reducing the complex viscosity of the PLA/P-PPD-Ph-conjugated flame-retardant composites and exhibiting shear thinning behavior. Therefore, the PLA/P-PPD-Ph-conjugated flame-retardant composites have a droplet phenomenon when burning, and the droplet takes a large amount of heat, thus obtaining a better combustion grade of the PLA/P-PPD-Ph-conjugated flame-retardant composites.

**FIGURE 2:**
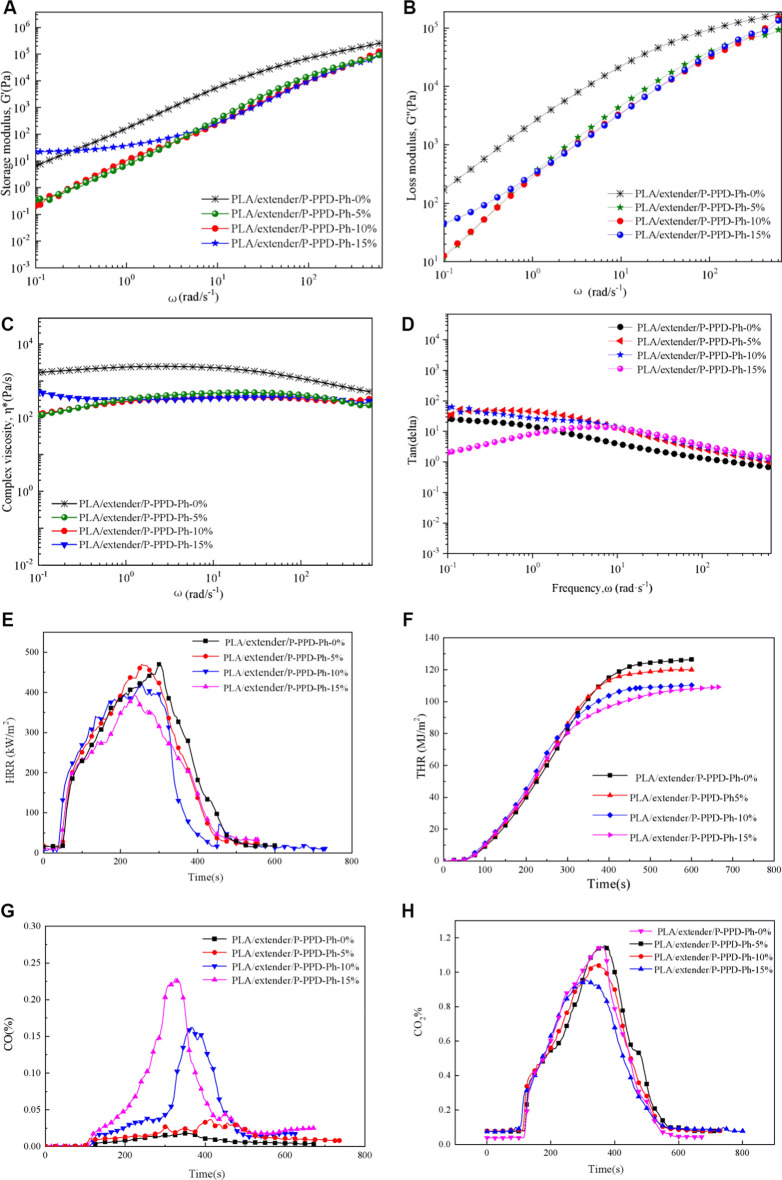
Storage modulus **(A)**, loss modulus **(B)**, complex viscosity **(C)**, and Tan(delta) **(D)** vs angle frequency for the PLA/P-PPD-Ph-conjugated flame-retardant composites at 200°C and cone calorimeter test of PLA/P-PPD-Ph-conjugated flame-retardant composites; **(E)** HRR curves, **(F)** THR curves, **(G)** CO release curves, **(H)** and CO_2_ release curves.

To further analyze the effect of P-PPD-Ph-conjugated flame retardant on the combustion behavior of PLA/P-PPD-Ph-conjugated flame-retardant composites, a cone calorimeter test of PLA/P-PPD-Ph-conjugated flame-retardant composites was performed. The test results are presented in Figures 2E–H; Table 2. Compared with PLA, the addition of P-PPD-Ph-conjugated flame retardant reduces TTI; this is probably because the thermal stability of P-PPD-Ph-conjugated flame retardant is relatively lower than that of PLA, and the addition of P-PPD-Ph-conjugated flame retardant reduces the thermal stability of the PLA/P-PPD-Ph-conjugated flame-retardant composites. P-PPD-Ph with 5 wt% addition has little effect on PHRR, and 10 wt% and 15wt% P-PPD-Ph reduced the PHRR values of PLA/P-PPD-Ph-conjugated flame-retardant composites by 9.1 and 16.28%, respectively, and t_p_ decreased with the addition of P-PPD-Ph. This further confirms that the addition of P-PPD-Ph reduces the thermal stability of the PLA-conjugated flame-retardant composites. The addition of P-PPD-Ph-conjugated flame retardant reduced the THR value, and as the amount of P-PPD-Ph-conjugated flame retardant increased, the more the THR value decreased; the THR value decreased by 14.9% when the P-PPD-Ph-conjugated flame retardant was 15 wt%. Moreover, the addition of P-PPD-Ph-conjugated flame retardant greatly increases the TSR value of the PLA/P-PPD-Ph-conjugated flame-retardant composites. In combination with Figure 2G, the CO production rate is also significantly increased due to the phosphorus-containing compounds, which the P-PPD-Ph-conjugated flame retardant releases, and these compounds can inhibit flames and thereby increase the number of components that cause incomplete combustion. EHC expresses the contribution of the active constituents of the material to the heat release in the gas phase during combustion (Qian, et al., 2015); as the amount of P-PPD-Ph-conjugated flame retardant increased, av-EHC gradually decreased, indicating that the content of effective combustion components in the gas phase component decreased. Therefore, the decrease in EHC and a significant increase in TSR and CO production suggest that the quenching activity of these compounds in PLA/P-PPD-Ph-conjugated flame-retardant composites mainly involves gaseous flame inhibition. The gas-phase flame-retardant mechanism is mainly due to the phosphorus-containing free radicals released during the decomposition of P-PPD-Ph-conjugated flame retardant, and these phosphorus-containing free radicals can capture free radicals such as H·, O·, or HO· in the flame combustion region to suppress the flame. Furthermore, PLA/P-PPD-Ph-conjugated flame-retardant composites produce more CO and less CO_2_ than pure PLA during combustion. Among the compounds, with the increasing amount of P-PPD-Ph-conjugated flame retardant added, the amount of CO generated is increased, and CO_2_ is reduced, and this means the addition of a flame retardant suppresses the combustion of the composites resulting in the flame-retardant effect of incomplete combustion, thus increasing the release of CO and reducing the release of CO_2_ in combustion fumes and gas phase.

**TABLE 2:** Cone calorimeter test data of PLA/P-PPD-Ph-conjugated flame-retardant composites.

Sample	TTI	PHRR (KW/m^2^)	t_p_ (s)	MAHRE (KW/m^2^)	THR (MJ/m^2^)	Av-HRR (KJ/m^2^)	Av-EHC (MJ/kg)	TSR (m^2^/m^2^)
PLA/extender/P-PPD-Ph-0%	39	470	300	292	126	225	16	3.7
PLA/extender/P-PPD-Ph-5%	38	467	265	295	120	232	15	803.8
PLA/extender/P-PPD-Ph-10%	35	427	255	250	116	195	15	1,127.5
PLA/extender/P-PPD-Ph-15%	31	393	235	283	107	124	14	1,573.4

TTI, time to ignition; PHRR, the peak of the heat release rate; t_P_, time at the peak of heat release rate; MAHRE, maximum average rate of the heat emission value; THR, total heat release; Av-HRR, average heat release rate; Av-EHC, average effective heat of combustion; TSR, total smoke release.

## Conclusion

The P-PPD-Ph-conjugated flame retardant was successfully synthesized by FTIR, ^1^H, and ^31^P NMR analysis. The T_−5%_ of P-PPD-Ph-conjugated flame retardant was 74°C higher than the T_−5%_ of DOPO, and the carbon residue at 800°C was increased by 25.8%. The PLA/P-PPD-Ph-conjugated flame-retardant composites have lower storage modulus, loss modulus, and complex viscosity parameters. PLA/P-PPD-Ph-conjugated flame retardant affects PLA/P-PPD-Ph-conjugated flame-retardant composites for promoting the formation of the carbon layer, when the P-PPD-Ph content was 15% and the residual carbon ratio for PLA-conjugated flame-retardant composites increased by 4.2%. As the amount of flame retardant is increasingly added, the PHRR value also decreases.

## Data Availability

The original contributions presented in the study are included in the article/Supplementary Material; further inquiries can be directed to the corresponding authors.
